# Dual PCM integrated thermoelectric generator for harvesting energy from ambient temperature variations

**DOI:** 10.1039/d6ra02643e

**Published:** 2026-04-21

**Authors:** Swathi Krishna Subhash, Jesvin Jose, Peter Woias, Uwe Pelz

**Affiliations:** a Department of Microsystems Engineering – IMTEK, University of Freiburg Germany swathi.subhash@imtek.uni-freiburg.de; b Cluster of Excellence livMatS @ FIT – Freiburg Center for Interactive Materials and Bioinspi Technologies, University of Freiburg Germany

## Abstract

Traditional thermoelectric generators (TEGs) rely on external heat sources and heat sinks to maintain a thermal gradient and generate electrical power. Here, we present a dual phase change material (PCM) integrated TEG system that generates an internal temperature gradient without a defined hot or cold side. Two PCMs with different melting points are positioned on opposite sides of the TEG, enabling the system to create repeatable thermal hysteresis during heating and cooling cycles. To improve the thermal conductivity of the PCM, we developed a hybrid composite PCM containing graphene nanoplatelets and copper metal wool, achieving a thermal conductivity of 0.98 W m^−1^ K^−1^ while maintaining a latent heat of 160–170 J g^−1^. Prototype testing under ambient-like temperature variations demonstrated stable and repeatable voltage generation, with open-circuit voltages exceeding 10 mV for 100–130 minutes. Composite PCMs provided faster and more uniform thermal responses than pure PCMs, enabling more predictable output. This dual PCM-TEG harvester represents a novel operational concept of passive energy harvesting, enabling conversion of low-grade, non-directional ambient thermal fluctuations into electrical power. The approach is well-suited for low-power autonomous sensors and IoT devices where external thermal gradients are unavailable.

## Abstract 2

A novel dual-PCM thermoelectric generator that exploits mismatched phase-change kinetics to internally generate a temperature gradient, enabling sustained electrical energy harvesting from ambient temperature variations like day–night cycles.

## Graphical abstract

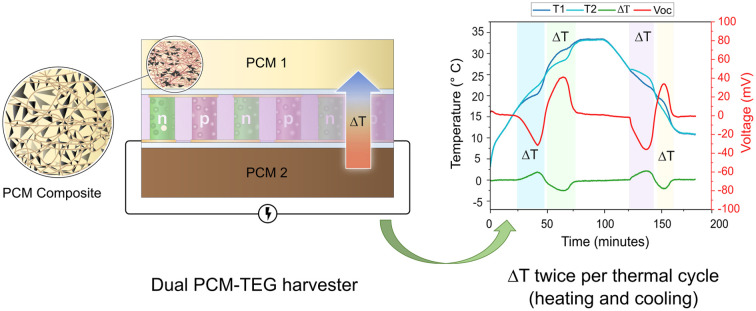

## 1 Introduction

Renewable energy harvesting and green energy production are usually considered in the context of large-scale applications with high energy demand. However, this focus overlooks the potential of thermoelectric generators (TEGs) for harvesting small amounts of ambient heat, which is advantageous for applications prioritizing autonomy and durability.^1^ TEGs are energy harvesters that can harvest energy from temperature gradients and convert it directly into electricity based on the Seebeck effect. Traditional TEGs are directional devices, with a hot side (heat source) and a cold side (heat sink), where heat flows unidirectionally from hot to cold. The greater the temperature difference between these two sides, the greater the voltage generated. In ambient settings where the entire device experiences the same fluctuating temperature, the thermal gradient collapses, resulting in low or intermittent energy production.

To mitigate this issue, TEGs are often equipped with additional heat sinks like rigid metal fins or spreaders or cooling fans that require additional cooling energy.^2^ Phase change materials (PCMs) offer a promising passive approach to stabilizing thermal gradients because they absorb and release latent heat at constant phase transition temperatures.^3^ This latent heat energy is widely used in thermal storage and management applications^4–12^ and is often integrated with TEGs.^13^ Unlike conventional heat sinks, PCMs can also store a significant amount of heat during phase transitions, thus maintaining a stable temperature gradient. When the temperature drops, they release the stored heat, providing more uniform thermal conditions across the TEG.

There are three possible positions for the PCM in relation to the TEG: PCM on the hot side, PCM on the cold side, and PCM on both sides. Previous studies have shown that applying PCMs to the hot side of a TEG module helps to harness energy from fluctuating temperatures and protects the module from overheating due to high heat loads, which usually damage the system when applied instantaneously.^14^ Other studies have explored the use of PCMs as effective heat sinks for TEGs. These studies showed that a PCM on the cold side of the TEG helped to maintain higher temperature differentials,^15^ as these PCMs could later act as a heat source (when the heater was turned off or during the night), granting an extended energy generation time. Nevertheless, single PCM configurations create directional thermal behavior, limiting placement flexibility and reducing effectiveness under uniform ambient heating.^16^

Three seminal papers have reported on the dual PCM-TEG configuration, where a TEG is sandwiched between two different PCMs. Atouei *et al.* experimentally demonstrated that combining PCMs on both sides stabilises temperature fluctuations and improves the overall voltage generation, though it reduces the maximum voltage. This makes the setup suitable for wireless sensor networks.^14^ Borhani *et al.* highlighted that incorporating copper porous media with PCM on both sides reduces voltage fluctuations and improves thermal conductivity.^17^ Yousefi *et al.* also demonstrated a system using PCM as energy storage on the hot side and a zero-energy cooling system on the cold side. Combined with copper foam, the dual PCM system prolongs energy generation while preventing voltage drop compared to the pure PCM system due to the reduced thermal resistance in the PCM system.^18^ These studies all highlight the benefits of using PCMs on both sides of the TEG for overall performance improvement by mitigating temperature fluctuations and extending operating time.

Despite the demonstrated benefits of dual-sided PCM arrangements, a significant research gap remains: all previous dual PCM-TEG studies rely on a clearly defined hot and cold side, generated by asymmetric heating, forced convection, or heat sinks connected to external sources. There is a critical lack of investigation into PCM-TEG systems operating under uniform, non-directional, low-grade ambient thermal fluctuations. To address this gap, the primary objective of this study is to design, construct, and evaluate a dual PCM-TEG harvester capable of capturing electrical energy entirely from simulated ambient temperature variations (analogous to the natural thermal variations of a day–night cycle). The fundamental novelty of this work lies in exploring this dual PCM configuration without any external heat source or sink, where a temperature differential is generated internally within the PCM-TEG assembly. Unlike earlier dual PCM studies, our system is subjected to a uniform, non-directional external temperature profile, and the gradient arises solely from the mismatched thermal responses of two PCMs with distinct melting points placed on opposite sides of the TEG. This enables the system to create two distinct temperature gradients per thermal cycle, during both the heating and cooling phases.

In addition to introducing this novel operational concept, this study also investigates the underexplored influence of high thermal conductivity composite PCM, on dual PCM-TEG performance. Several studies have explored different strategies to overcome the low thermal conductivity of PCMs. Among them, a primary method is the inclusion of nanoparticles^19^ such as carbon nanotubes,^20^ graphene nanoparticles,^21^ and nanoplatelets^22^ or metallic nanoparticles.^23^ Recent studies have also studied hybrid metal–carbon mixtures to improve TC enhancement without compromising the stability of the PCM composite. For example, Bhutto *et al.* dispersed hybrid silver-graphene nanoparticles into lauric acid for TEG applications. They reported that a 3.5 wt% addition improved the PCM's TC by 57% (up to 0.314 W m^−1^ K^−1^), which allowed the nanocomposite-enhanced TEG to generate a higher peak voltage and achieve a faster thermal response compared to the pure PCM system. However, they still identify the limitation of aggregation and clustering of nanoparticles at higher concentrations, which negatively impacts thermal conductivity.^24^

To address both TC and structural stability without relying solely on suspended nanoparticles, an alternative technique utilizes highly conductive macroscopic scaffolds or porous foams of carbon-based materials^25^ or metals.^17^ These porous scaffolds can hold the PCM, providing critical form-stability to prevent liquid leakage, and at the same time enhance TC by establishing continuous heat conduction pathways. However, high-volume scaffolds can proportionally reduce the overall latent heat capacity, which directly diminishes their management capabilities.^25^

To overcome the respective limitations of isolated nanoparticles and bulk scaffolds, recent hybrid approaches synergistically combine conductive nano-additives with macroscopic matrices. For instance, Yu *et al.* demonstrated that combining single-walled carbon nanotubes (SWCNTs) within a highly porous metal foam skeleton dramatically accelerates melting rates, enhances TC without drastically reducing latent heat, and ensures uniform temperature distribution.^26^

In this context, the present study utilized a hybrid metal-wool/graphene-nanoplatelet (GnP) additive to improve heat transfer and dissipation within the PCM and to/from its thermal interfaces. Specifically, GnP is dispersed within a percolating macroscopic copper wool network to create efficient, stable heat-transfer pathways. The thermal and morphological properties of these composites are characterized in this work and are tested for thermal management in TEGs. A direct comparison between pure and composite PCMs under identical thermal excitation using both narrow and wide melting range pairs was performed under slow, ambient-like heating and cooling conditions. The resulting system performance was evaluated by monitoring the temperatures on both sides of the TEG and the open-circuit voltage of a commercial TEG module, providing new insights into the trade-off between latent heat, thermal kinetics, cycle speed, and voltage stability.

## 2 Design and concept of dual PCM-TEG harvester

**Fig. 1:**
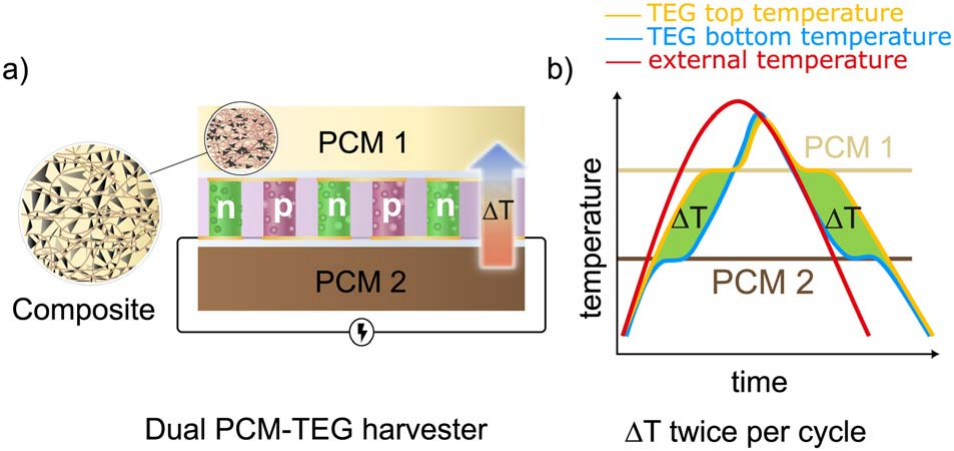
Schematic of the proposed dual PCM-TEG harvester. (a) Cross-sectional view of the system, showing a thermoelectric generator (TEG) with n- and p-type thermolegs, sandwiched between two distinct PCMs, each doped with copper wool and graphene nanoplatelets (GnP). (b) Working principle of the system as an ambient temperature-driven energy harvesting unit. The red curve represents ambient temperature fluctuations, while the blue and yellow curves indicate temperature variations across the TEG.

In the proposed system, a commercial TEG is placed between PCMs with different melting temperatures. When the external environment warms or cools uniformly, each PCM responds differently due to its specific phase transition temperature range. This mismatch in thermal response generates an internal temperature gradient across the TEG, even though both sides experience the same external temperature. The schematic of the proposed system is shown in Fig. 1(a). The TEG is sandwiched between PCM1 and PCM2, each doped with GnP and copper wool to enhance thermal conductivity.

Two PCM combinations were examined: narrow melting range pair (21 °C and 29 °C) and a wider melting range pair (15 °C and 32 °C). A wider melting range pairing can support broader operating conditions and may provide a longer phase change duration, enabling an extended operating period. Conversely, narrow melting-range pair may allow faster and more distinct switching in phase change, and a more instantaneous voltage generation. Studying these two configurations helps us to understand the performance characteristics and choose the melting ranges according to the intended application.

Fig. 1(b) illustrates the mechanism: as the ambient temperature rises (red curve) and approaches the melting point of PCM2, PCM2 begins to melt (blue curve), while PCM1 continues heating (orange curve). In this phase, a transient temperature gradient is produced across the TEG, and heat flows from PCM1 to PCM2. When the ambient temperature reaches the melting range of PCM1, it also melts at a constant temperature (yellow curve), and a second plateau forms. Once both PCMs are liquid, the temperature difference across the TEG falls to zero. During cooling, the process reverses: PCM1 solidifies first while PCM2 follows the ambient temperature, again establishing a temporary thermal gradient. This heating–cooling cycle produces a hysteresis effect that maintains a temperature difference across the TEG for limited periods in both directions. The design eliminates the need for heat sinks or directional heat inputs, allowing the system to operate under ambient thermal fluctuations such as diurnal cycles, indoor temperature variations, or intermittent low-grade waste-heat exposure.

## 3 Experimental methods

### 3.1 Preparation of PCM/carbon-materials composites

Paraffin PCMs with melting temperatures of 15 °C, 21 °C, 29 °C, and 32 °C (Croda Ibérica SAU, Spain) were used. The latent heat values were in the range of 180–210 J g^−1^ as determined by differential scanning calorimetry (DSC) according to the supplier's data sheet. Two carbon additives were investigated: µGr (ProGraphite GmbH) and graphene nanoplatelets (GnP, Nanografi), due to their high intrinsic thermal conductivity. Sodium dodecyl benzene sulfonate (SDBS, Sigma-Aldrich) was used as a stabilising agent to improve the dispersion of carbon additives. Copper wool (Carl Roth GmbH) served as a metallic conductivity enhancer.

**Fig. 2:**
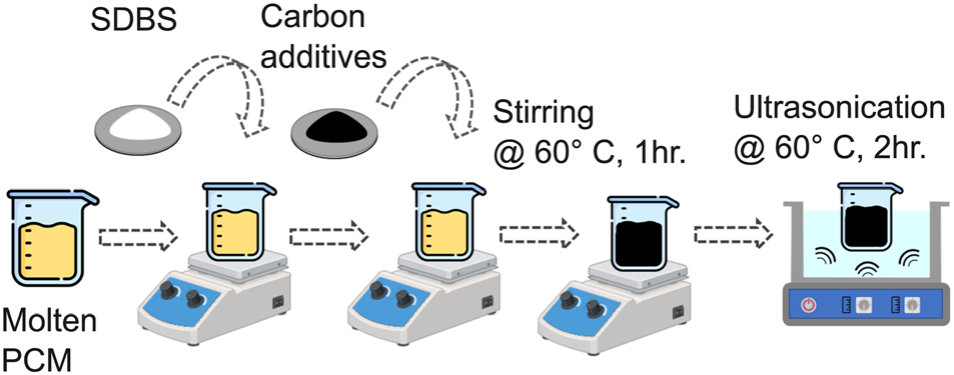
Two-step dispersion process of composite PCMs.

The PCM composites were prepared by a two-step dispersion process, as shown in Fig. 2. Paraffin was melted at 60 °C and mechanically stirred. Carbon additives (1, 2, and 3 wt%) were incorporated and mixed, followed by ultrasonication (Sonorex Digiplus DL 102 H, 35 kHz, 120/480 W, Bandelin) at 60 °C to achieve homogeneous dispersion. Samples with and without SDBS were prepared to evaluate surfactant influence. Final high-conductivity composites incorporated 3 wt% GnP along with 2 vol% copper wool, which acts as a porous conductive scaffold.

### 3.2 Characterization

The thermal conductivity of the composites was measured using the transient hot-bridge system (THB Advance, Linseis, Germany) with a THB A sensor. For each sample, measurements were repeated 10 times, and the reported values represent the mean ± 0.01 standard deviation. Differential scanning calorimetry (DSC, NETZSCH 204F1) was used to determine melting range, latent heat, and thermal stability, at a flow rate of 20 mL min^−1^, a heating rate of 5 °C min^−1^, and within a temperature range of 0–60 °C. To ensure repeatability, standard thermal property measurements were averaged over 5 consecutive heating–cooling cycles, while thermal stability was assessed over 30 cycles. The instrument provides a temperature accuracy of ±0.01 °C and an enthalpy accuracy typically better than 1%. SEM (FEI Scios 2 Hivac) and FTIR (Agilent Cary 630) were used to characterize the microstructure and chemical stability of the composites; results are provided in SI (Fig. S1). Infrared thermography (FLIR A65) visualized heat transfer uniformity during heating and cooling in PCMs with and without thermal additives. The samples were heated using a hot plate at 60 °C simultaneously, and the temperatures were recorded.

### 3.3 Experimental setup

**Fig. 3:**
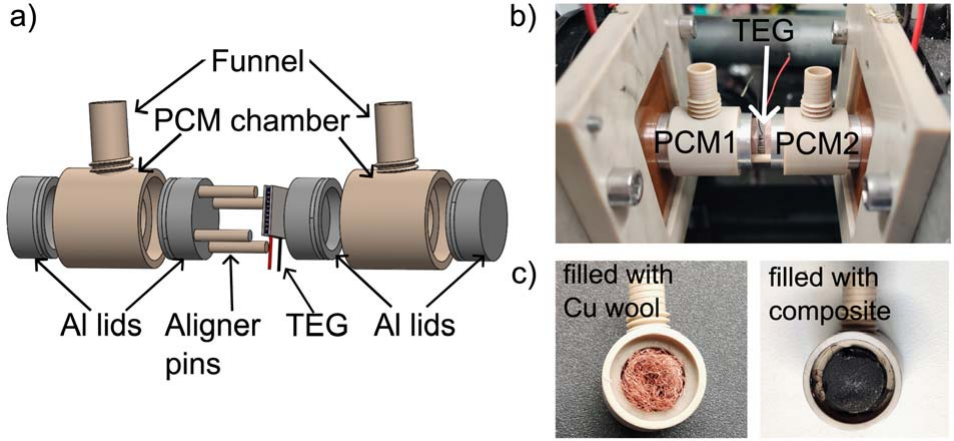
Schematics and photograph of the experimental setup. (a) Solidworks schematics of the dual PCM-TEG harvester containing two PCM containers with aluminium lids and a TEG sandwiched between them using aligner pins. (b) Photograph of the dual PCM-TEG harvester sandwiched between the copper plates in the custom-built temperature controller called TACS. (c) PCM container filled with Cu wool (left) and later the final PCM metal wool-carbon material composite (right), which was used for the performance experiments.

A dual PCM-TEG harvester prototype was designed and constructed. The exploded view of the dual PCM-TEG harvester is shown in Fig. 3(a). The device consists of a commercial TEG module (1MC06-048-15_TEG, 48 thermolegs, 4.28 Ω internal resistance, 10 mm × 10 mm) positioned in the center and two cylindrical PCM containers on either side housing two different PCMs.

The lateral surfaces (*i.e.*, the side walls) of the containers were made from PEEK (polyether ether ketone), a material with relatively low thermal conductivity (∼0.25–0.4 W m^−1^ K^−1^) to minimize lateral thermal loss. The top and bottom faces of the container were made of aluminium lids that conducted the heat from the PCM to the TEG. To accommodate the volume expansion of the PCM during the solid–liquid phase change, the container was equipped with a piston at the top. The total volume of one container is 3.5 mL.

The dual PCM-TEG harvester was placed between two temperature-controlled copper plates within a custom thermal adjustment and control setup (TACS) (see Fig. 2(b)). The copper plates were precisely temperature-controlled using Peltier elements (see ref. 27 for the detailed description of the setup). During measurement, both plates were synchronously heated/cooled to impose a uniform external temperature profile. Temperatures at both TEG interfaces were recorded using T-type thermocouples at 20-s intervals. Open-circuit voltage was measured using a Keysight 34465A multimeter, with a sampling rate of 1 sample per 20 seconds. Fig. 3(c) shows photographs of the container first filled with only Cu wool and then filled with a composite PCM and Cu wool.

## 4 Results and discussion

### 4.1 Thermal properties

Paraffin PCMs typically exhibit low thermal conductivity (TC) values (0.17–0.24 W m^−1^ K^−1^), which limit their ability to distribute heat uniformly and affect phase change behaviour. Many studies in the literature have reported the use of carbon-based additives such as graphite,^28^ graphene oxide,^29^ graphene nanoplatelets (GnP),^22^ and expanded graphite,^30^ as TC enhancers for PCM. In this study, to improve the thermal conductivity, micro-graphite (µGr) and graphene nanoplatelets (GnP) were incorporated at 1–3 wt% loading, with and without SDBS surfactant.

**Fig. 4:**
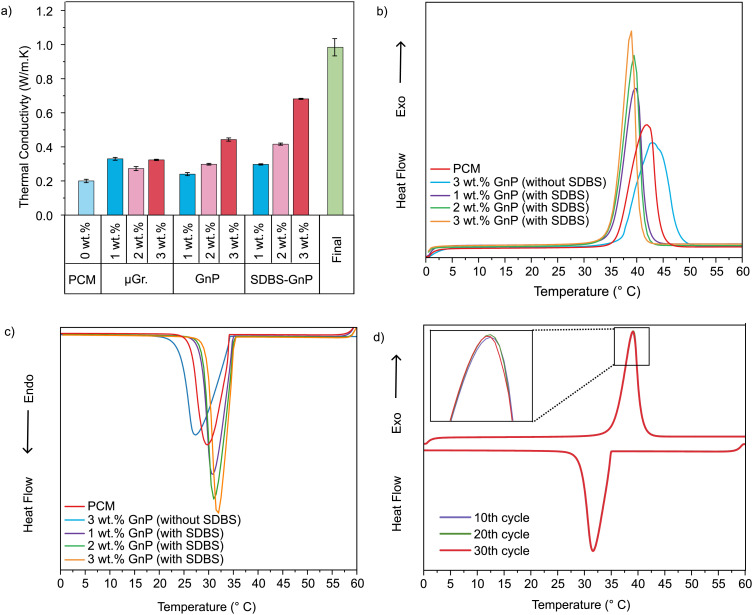
(a) Thermal conductivity of PCM, PCM-µGr. Composite (1, 2, 3 wt%), PCM-GnP composite (with and without SDBS (1, 2, 3 wt%), and final composite with 3 wt% GnP and 2 v/v % Cu wool. (b) DSC heating curve of PCM, 3 wt% GnP composite without SDBS, and GnP composites with SDBS (1, 2, 3 wt%) showing the effect of SDBS and the increasing weight percentage of filler. (c) Corresponding DSC cooling curve for the sample. (d) DSC thermal cycling curve of PCM-GnP composite (3 wt%, with SDBS) at 10th, 20th, and 30th cycle of heating and cooling.

The thermal conductivity of paraffin composites measured as a function of loading with different carbon additives (µGr and GnP in 1–3 wt%) along with pure PCM is shown in Fig. 4(a). The optimal thermal conductivities for the two materials were obtained at a 3 wt% loading, achieving 0.32 and 0.44 W m^−1^ K^−1^ for µGr and GnP, respectively. However, these samples displayed very poor stability, with the particles separating within 24 h.

As a uniform distribution of materials is vital for TC enhancement,^31^ SDBS was used to improve the dispersion of GnP (chosen for its stronger TC enhancement properties) in the composite. After the addition of SDBS, the samples showed a profound and linear improvement in TC compared to those without SDBS. The addition of SDBS ensures the GnP is dispersed completely and uniformly throughout the PCM. This homogeneous dispersion provides greater interaction between the PCM matrix and GnP, offering more particles available for heat transfer, thus enhancing thermal conductivity.^32^ The maximum value measured for these samples was 0.68 W m^−1^ K^−1^ in the 3 wt% sample, corresponding to an enhancement of more than 240% compared to pure paraffin. The improved performance can be attributed to the formation of a suitable GnP network, which reduces interfacial thermal resistance. These measured values fall within the upper range of those previously reported for paraffin-GnP composites with a 3 wt% filler content.^33,34^

Previously, we have reported copper wool as a TC enhancer for PCMs, achieving around 0.49 W m^−1^ K^−1^ (+145% in TC) with the addition of less than 3 v/v% of copper wool. The Cu wool creates a percolating conductive network that increases the conduction and suppresses the convective currents in molten PCM.^27^ Building on this concept, the present work incorporates 2 v/v % Cu wool together with 3 wt% GnP in a final composite. The wool acted as a wick, preventing the partial settling of the graphene nanoplatelets and further improving the stability of the dispersion, especially in the liquid state. The TC of this resulting composite reached 0.98 W m^−1^ K^−1^, corresponding to a 390% overall TC enhancement over paraffin alone. Although the individual benefits of GnP and copper wool as thermal conductivity enhancers are well established, to the best of our knowledge, their combined use in a single paraffin composite has not been examined. This work, therefore, presents and evaluates this hybrid enhancement strategy.

The results of the thermal conductivity measurements showed that the GnP composite produced the best values. Therefore, further characterisations focused solely on this composite. Fig. 4(b and c) shows the DSC curves of the composite samples and confirms that all composites preserve the PCM's characteristic melting and solidification behaviour. Increasing GnP concentration sharpened melting/solidification peaks and narrowed the transition interval, indicating faster heat transfer and reduced supercooling. A comparison of the DSC peaks for 3 wt% samples with and without SDBS highlights how SDBS improves the dispersion quality. The peaks of the samples without SDBS were even broader than those of pure PCM, indicating ineffective local heat transfer through the composite, despite the presence of a highly thermally conductive additive. The composite also demonstrated excellent thermal stability over 30 heating–cooling cycles, with no significant change in melting range or enthalpy as seen in Fig. 4(d).

The key parameters extracted from the DSC analysis, the melting range, heat storage enthalpy (heating/cooling), and the corresponding thermal conductivity, are listed in Table S1 (SI). Latent heat decreased only modestly (from 185.5 J g^−1^ to 170.5 J g^−1^ at 3 wt% GnP with SDBS). This represents an 8% reduction in latent heat, which is a very respectable value, alongside a 284% increase in TC compared to pure PCM (0.68 W m^−1^ K^−1^*versus* 0.24 W m^−1^ K^−1^).

**Fig. 5:**
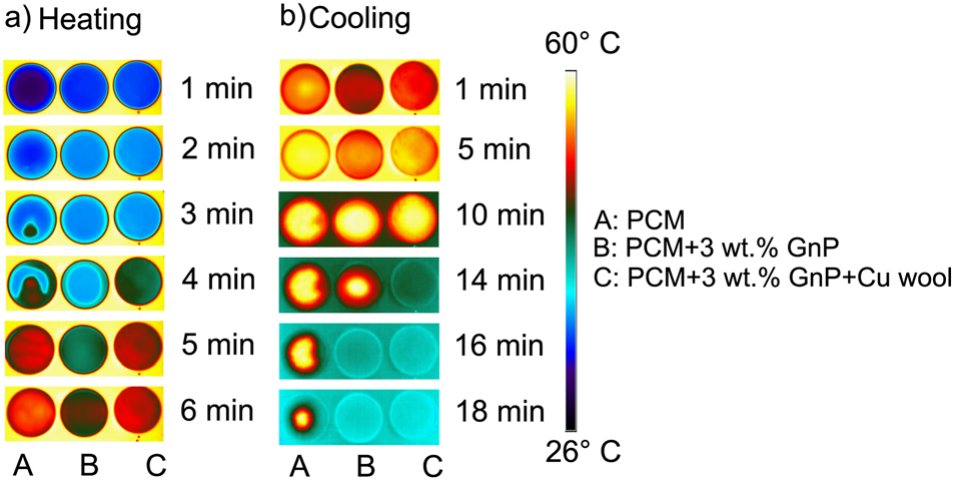
Infrared images of PCM during a heating and cooling cycle; PCM–GnP composite and PCM–GnP–Cu wool composite at different time intervals (a) when heated on a hotplate at 60 °C for 10 min. (b) While passively cooling after the hotplate was switched off.

The infrared images of pure PCM (A), PCM–GnP composite (B), and PCM–GnP–Cu wool (C) composite at various time intervals during heating and cooling are shown in Fig. 5(a) and (b), respectively. During heating, the pure PCM melted unevenly, forming localized liquid pockets and exhibiting clear phase separation. This promoted internal convection, giving the appearance of faster heating, but resulted in non-uniform heat distribution. This non-uniformity persisted during cooling, where portions of the PCM remained liquid even after extended periods, indicating poor reversibility.

In contrast, both the PCM–GnP and PCM–GnP–Cu wool composites exhibited uniform melting and solidification with no visible phase separation. Heat propagated smoothly from the outer edges toward the core, producing a homogeneous temperature field. The PCM–GnP–Cu wool composite showed the fastest thermal response due to the conductive, porous copper wool network. These results confirm that the composite, particularly the Cu-enhanced version, provides more efficient, repeatable, and reversible heat transfer, making it the most suitable choice for thermal energy-storage applications.

### 4.2 Dual PCM-TEG harvester performance

The dual PCM-TEG system performance was tested under a single heating–cooling cycle and over three consecutive heating–cooling cycles to evaluate the thermal stability. An ‘active energy harvesting’ is defined as a period during which the TEG's open-circuit voltage exceeds 10 mV. This 10 mV threshold was selected to capture the full functional operating window of the TEG during low-gradient PCM phase transitions. This value is consistent with the operating limits of current ultra-low power management systems, particularly the specialized converter architectures developed within our own research group. For instance, Woias *et al.* demonstrated a self-supplied low-voltage boost converter combining a Meissner oscillator and a forward converter that initiates resonant operation at just 6.2 mV and robust forward conversion at 10 mV, achieving peak power conversion efficiencies exceeding 30%.^35^ Advancing further in this direction, our group's most recent symmetric boost converter designs successfully achieve active thermoelectric energy harvesting from input voltages as low as 6 mV with a 66% peak efficiency.^36^ While these in-house capabilities are also corroborated by broader literature validating the 5 to 10 mV (ref. 37 and 38) operational range for modern DC–DC converters, our group's specific hardware developments confirm that a 10 mV threshold represents a highly practical, demonstrable lower bound. By setting the threshold at 10 mV, we ensure that the reported harvesting periods accurately reflect the true, achievable capacity of the system to drive autonomous IoT devices. The voltage values reported are the average value over this active period. Experiments were conducted using narrow melting range PCM pairs (21/29 °C) and wider melting range PCM pairs (15/32 °C) using pure PCM and thermally enhanced composite PCMs (3 wt% GnP + 2 vol% Cu wool) to study how melting point separation, latent heat capacity, and thermal conductivity influence the internal gradient.

#### 4.2.1 Narrow melting range PCM pairs

**Fig. 6:**
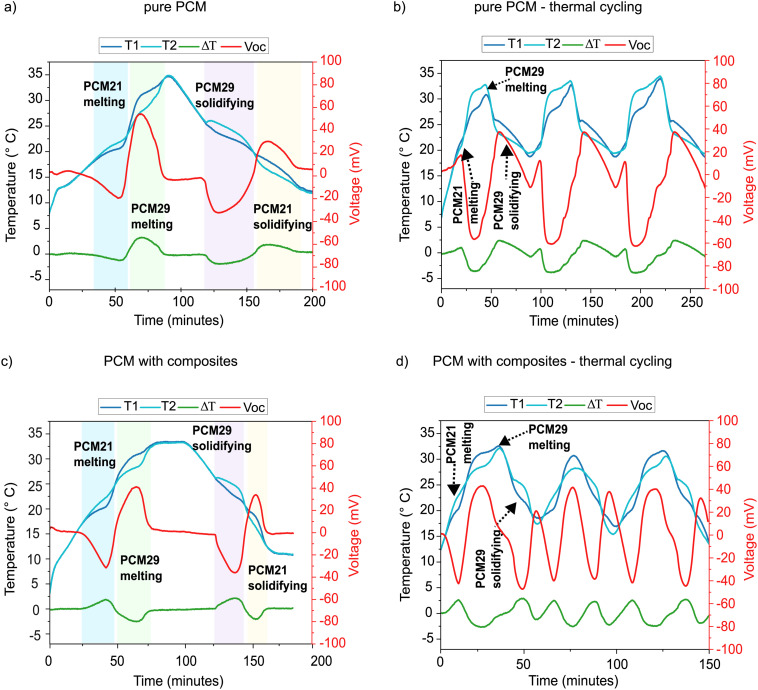
Dual PCM-TEG harvester performance measurement with narrow melting range of PCM (21 °C and 29 °C). Temperature *vs.* time profile and corresponding TEG open circuit voltage *vs.* time profile of PCM21 and PCM29 tested with and without composites on either side of the TEG during a cycle of controlled heating and cooling, and during thermal cycling (thermal transient load). T1 (dark blue curve) is the temperature measured at the TEG face in contact with the PCM21 and T2 (light blue curve) is the temperature measured at the TEG face in contact with PCM29. (a) Single cycle, pure PCM (b) thermal cycling, pure PCM (c) single cycle, PCM composite with 3 wt% GnP and Cu wool (d) thermal cycling – PCM composite with 3 wt% GnP and Cu wool.

For the narrow melting range, PCMs with melting points of 21 °C (PCM21) and 29 °C (PCM29) were selected. The temperature curves (measured on either side of the TEG), corresponding temperature difference across the TEG (Δ*T*), and the open circuit voltage (*V*_oc_) from the TEG are summarized in Fig. 6. The absolute values of the maximum Δ*T* and *V*_oc_ obtained during each melting/solidification process are listed in Table S2 (SI).

##### 4.2.1.1 Controlled heating and cooling (to mimic the ambient environment)

As seen in Fig. 6(a) for the pure PCM and (c) for the composite PCM systems, four distinct hysteresis regions were observed from the melting and solidification of the two different PCMs, and these regions are color-coded in the figures (refer to Fig. 1(b) for the theoretical explanation).

Region I (blue) – melting of PCM21: a first temperature plateau can be seen around 20 °C. Both the pure PCM and composite system showed a small Δ*T* of 1–2 °C. In the composite, however, PCM29 heated more rapidly, indicating a steeper temperature rise that can be attributed to the composite's higher effective thermal conductivity. During the phase transition, the pure PCM system produced an average voltage of 15 mV over a 15 min active energy harvesting, whereas the composite system produced 22 mV for approximately 16 min.

Region II (green) – melting of PCM29: this region produced the highest Δ*T* and maximum voltage in both systems. The pure PCM was harvested for about 25 min with an average output voltage of 39 mV. In the composite system, this was reduced to 23 min and 29 mV mainly due to lower overall heat capacity caused by replacing part of the PCM with the additives.

Region III (purple) – solidification of PCM29: during cooling, PCM29 released latent heat while PCM21 cooled quickly, forming a third thermal gradient. The cooling slopes were less steep in the pure PCM system compared to the composite. Both systems produced similar voltages of about 25 mV, however, the slow cooling slope of pure PCM enabled prolonged harvesting for about 30 min compared with 20 min for the composite system.

Region IV (yellow) – solidification of PCM21: this region exhibited a similar trend to Region III, and produced around 25 mV for both pure PCM and composite systems. However, the pure PCM sustained harvesting for about 28 min, whereas the composite lasted only about 12 min. The composite solidified faster due to improved heat conduction, shortening the overall cooling duration and completing the cycle sooner.

The thermal behaviour of the composite PCM system is distinctly different from that of the pure PCM system due to the effects of the additives. While the pure PCM system consistently achieved higher peak voltages, the composite system exhibited advantages in terms of thermal kinetics. The enhanced thermal conductivity of the composite created short heat pathways and promoted quicker and more uniform heating and cooling. This minimises supercooling and smooths the phase change fronts. As a result, the composite PCMs solidified more rapidly and completed the full thermal cycle noticeably faster despite their lower latent heat capacity. Comparing Fig. 6(a) and (c) highlights this difference: the pure PCM system took approximately 100 min to complete all four phase changes (active energy harvesting period), while the composite completed the process in 70 min, representing a 30% faster thermal response. It is also worth noting that, unlike the fluctuating output in the pure PCM (15–39 mV), the average voltage outputs (25 mV) over different phase change regions in the composites were stable. This stability, although not increasing total harvested energy, is advantageous for sensitive or regulated systems where predictable voltage is more valuable than maximum amplitude.

##### 4.2.1.2 Transient thermal cycling

The temperature and voltage profiles over three cycles of heating and cooling are shown in Fig. 6(b) for the pure PCM system and Fig. 6(d) for the composite system. In the pure PCM system, clear melting and solidification regions of PCM29 were consistently seen across all the cycles, confirming stable and repeatable transitions. PCM21 exhibited a shoulder peak during the initial melting stage of each cycle, likely due to a higher heating rate and insufficient thermal stabilisation, resulting in an incomplete temperature plateau. During cooling, PCM21 solidified only partially because the ambient temperature remained above its crystallisation point, limiting complete phase change. The average voltage output across the three thermal cycles was 33 mV, with an active harvesting window of approximately 60 min per cycle.

The composite PCM system, however, showed more uniform hysteresis for both PCMs, indicating faster and more homogeneous heat distribution. Although its active harvesting window was slightly shorter (∼40 min per cycle), it delivered a comparable average voltage of ∼30 mV. A key difference between the pure and composite systems is the total cycle duration. The pure PCM required 250 min to complete all three cycles, whereas the composite system completed the same sequence in 160 min, representing a 36% reduction in total cycle time. This reduction in time clearly demonstrates the enhanced thermal responsiveness of the composite, which also produced more symmetric and repeatable voltage profiles due to improved heat transfer. In the narrow melting range system, across both single-cycle and full thermal-cycling experiments, the results reveal the complementary advantages of pure PCM and composite PCMs under fluctuating thermal conditions. The pure PCM system achieved higher peak voltages and longer harvesting intervals, but at the cost of slower transitions and greater output variability. Composite PCMs deliver faster response times, smoother phase change behaviour, and more stable voltage output, but with reduced peak voltages and lower energy density. These characteristics make composites better suited for applications driven by rapidly changing ambient temperature cycles, while pure PCMs remain preferable when energy density is the primary objective.

#### 4.2.2 Wider melting range PCM pairs

**Fig. 7:**
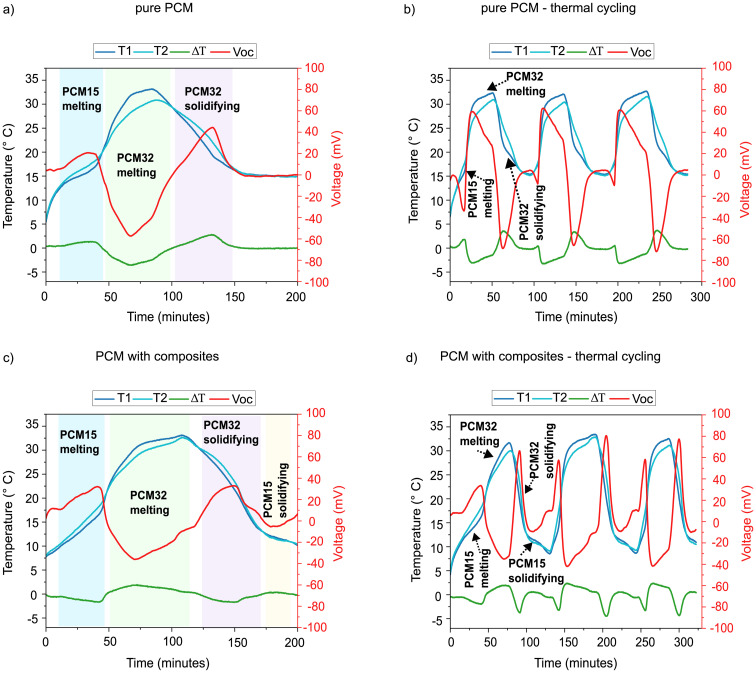
Dual PCM-TEG harvester performance measurement with wider melting range of PCM (15 °C and 32 °C). Temperature *vs.* time profile and corresponding TEG open circuit voltage *vs.* time profile of PCM15 and PCM32 tested with and without composites on either side of the TEG during a cycle of controlled heating and cooling, and during thermal cycling (thermal transient load). T1 (dark blue curve) is the temperature measured at the TEG face in contact with the PCM15, and T2 (light blue curve) is the temperature measured at the TEG face in contact with PCM32. (a) Single cycle, pure PCM (b) thermal cycling, pure PCM (c) single cycle, PCM composite with 3 wt% GnP and Cu wool (d) thermal cycling – PCM composite with 3 wt% GnP and Cu wool.

For the wider melting range, PCMs with melting points of 15 °C (PCM15) and 32 °C (PCM32) were selected. These PCMs span a temperature difference of 17 °C, enabling sustained thermoelectric output to be evaluated across a longer thermal ramp. The temperature curves (measured on either side of the TEG), corresponding temperature difference (Δ*T*), and the open circuit voltage (*V*_oc_) from the TEG are plotted and given in Fig. 7. The absolute values of the maximum Δ*T* and *V*_oc_ obtained during the melting/solidification process are listed in Table S3 (SI).

##### 4.2.2.1 Controlled heating and cooling (to mimic the ambient environment)

As seen in Fig. 7(a) for pure PCM and Fig. 7(c) for the composite systems, four hysteresis regions arising from the melting and solidification of two PCMs are present, and they are color-coded in the figures.

Region I (blue): melting of PCM15: in the pure PCM system, the hysteresis region was localised near the melting point of PCM15, resulting in a shorter active voltage generation window (∼25 min). The composite system generated an earlier and broader hysteresis region with a sustained Δ*T* for a longer time. This led to an energy harvesting of approximately 35 min. The average output voltage during this period is 17 mV and 23 mV for the pure PCM and composite systems, respectively.

Region II (green): melting of PCM32: this stage produced the most dominant and longest harvesting window. In the pure PCM system, supercooling in PCM32 caused phase separation and a temperature plateau near its melting point. Additionally, the temperature of PCM15 was buffered at 33 °C due to ambient heat losses in minimally insulated small PCM containers. The composite system exhibited a more effective heat transfer, allowing for a faster and complete melting of PCM32 and a smoother temperature equalisation. The pure PCM system produced an average voltage of 40 mV for about 47 min, while the composite system produced a lower voltage of 25 mV for about 56 min.

Region III (purple): solidification of PCM32: upon cooling, the release of latent heat from PCM32 formed the next hysteresis region. The composite system with enhanced thermal conductivity facilitated faster heat transfer. This resulted in an average voltage of 28 mV (for 37 min) for pure PCM and 23 mV (for 40 min) for the composite.

Region IV (yellow): solidification of PCM15: as can be seen in Fig. 7(a), this region is absent for pure PCM, as the system never reached a temperature below the melting point of PCM15, due to the effect of ambient temperature. However, in the composite, a small temperature difference was observed with mild hysteresis near the melting point of 15 °C. Even so, the temperature difference remained too small to generate more than ∼10 mV of voltage.

The composite PCMs maintained a stable average output voltage of about 25 mV across all phase transition regions, demonstrating consistency under dynamic thermal conditions. Their reduced peak voltages (as seen in Fig. 7(c)) stem from the dilution of latent heat capacity caused by the added copper wool, which lowers the energy released per transition. Despite this, the composite system achieved a longer harvesting window of 131 min *vs.* 109 min for the pure PCM. This extended harvesting duration in the composite arises from PCM hysteresis: the enhanced thermal conductivity allows the system to exploit the wider temporal separation between the two melting points more effectively, enabling sustained energy harvesting over a longer period of the cycle.

In contrast, the pure PCM showed larger Δ*T* than the composite system, driven by slower heating and cooling, which resulted in higher output voltages. However, the larger Δ*T* was created mostly because of supercooling, phase separation, uneven temperature equalisation, and extended plateaus near the melting points, all of which limited the pure PCM's ability to fully utilise the available thermal difference between the two PCMs.

##### 4.2.2.2 Transient thermal cycling

The temperature and voltage profiles obtained over 3 cycles of heating–cooling for pure PCM and composite system are shown in Fig. 7(b) and (d), respectively. These profiles are consistent single-cycle results (Fig. 7(a) and (c)), and were repeated reliably across three cycles.

In the pure PCM system, the dominant regions observed were the melting and solidification peaks of PCM32. PCM15 melted only during the first cycle, and was absent in the later cycles due to incomplete solidification after the first cycle. This occurred due to insufficient cooling, as the ambient temperature did not drop below the melting point of PCM15. Over the three thermal cycles, the active energy harvesting window was approximately 165 min with an average voltage output of 42 mV.

The same thermal cycling test was conducted with the composite system. Previous single-cycle experiments had shown that in the pure PCM system, PCM15 failed to solidify despite sufficient time. However, in the composite, the PCM15 showed a short region of solidification, indicating the potential to access this phase transition using composites. To facilitate the complete solidification of PCM15 in the composite system, an additional 10 min of heating and cooling were incorporated into the thermal cycling experiment. With this adjustment, the composite exhibited all four regions during both the melting and solidification of PCM15. The active energy harvesting period was about 210 min with an average voltage of 32 mV. This suggests that the improved thermal conductivity of the composite enabled access to the PCM15 phase transition. The additional duration in the composite cycling experiment was not intended to artificially enhance the performance, but rather to reveal the system's natural responsiveness in conditions that more closely resemble real-world thermal cycling scenarios. Full thermal cycling is crucial for reliable energy harvesting, and this experiment demonstrates that a composite system, with its improved thermal transfer, is capable of achieving this within a slight operational buffer.

In the wider melting range system, the composite system consistently showed a longer energy harvesting period than the pure PCM system. This improvement arises because the composites enabled access to additional phase transitions, specifically the solidification of PCM15, which was absent in the pure PCM system. Furthermore, the composites allowed effective utilization of the larger temperature differences within the PCM's melting range in the wider melting range setup, resulting in extended hysteresis regions. Although the composite produced a lower voltage output, it delivered stable voltage over longer periods, across all phase transition regions. Overall, in wider range systems, composite materials are more suitable for sustained and stable energy harvesting, especially in environmental or building-integrated applications.

The narrow melting range (PCM21/29) and wider melting range (PCM15/32) systems each offer unique advantages for thermal energy harvesting. In both of these scenarios, pure PCM consistently showed higher voltage output up to 54 mV in the narrow-range and 57 mV in the wider range. To evaluate the practical driving capability of the system, the theoretical maximum power output (*P*_max_) and power density were calculated based on the maximum power transfer theorem 
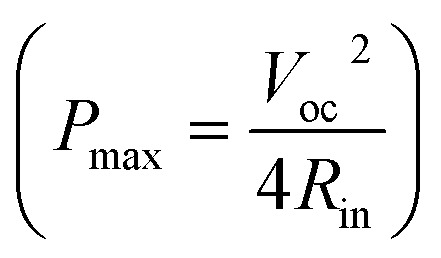
, as summarized in Table 1. Given the TEG's internal resistance of 4.28 Ω and area of 1 cm^2^, the peak power densities from the pure PCM configurations range from 170–190 µW cm^−2^, while the composite systems range between 75 and 98 µW cm^−2^.

**Table 1:** Maximum power output (*P*_max_) and power density of dual PCM-TEG systems (calculated based on *R*_in_ = 4.28 Ω and TEG area = 1 cm^2^)

System configuration	Material (PCM)	Peak *V*_oc_ (mV)	*P*_max_ (µW)	Power density (µW cm^−2^)
Narrow range (PCM21/29)	Pure	54	170.3	170.3
	Composite	41	98.2	98.2
Wider range (PCM15/32)	Pure	57	189.8	189.8
	Composite	36	75.7	75.7

However, since our dual PCM TEG system operated under slow ambient fluctuations, we have a transient energy harvesting system. Therefore, the true operational capability of the system is best represented by its sustained average voltage and total energy yield. Over a single heating–cooling cycle (during the active harvesting period), the systems were able to sustain 32–42 mV over periods of 165–210 minutes. Consequently, the pure PCM systems yielded substantial amounts of energy, delivering 306 J (PCM21/29) and 446 J (PCM15/32), compared with 200 J and 304 J from the respective composite systems.

This sustained energy supply over long periods of time is sufficient to drive realistic low-power loads. Recent literature demonstrates that specific ultra-low-power electronics, such as intermittent Bluetooth Low Energy (BLE) nodes equipped with temperature and current sensors, require only 2.4 Joules per hour to maintain 10–15 data transmissions.^39^ Furthermore, specific commercial environmental sensors (*e.g.*, BME280) integrated into MCU-free architectures require as little as 5 nanojoules per sample.^40^ Therefore, the total energy strictly harvested above our 10 mV threshold vastly exceeds the requirements to continuously power these modern autonomous IoT condition-monitoring sensors.

While the pure PCM configurations clearly maximize this total energy yield, these benefits were at the cost of slower thermal response, resulting in long cycle duration and fluctuating voltage output. Also, the high voltage peaks in pure PCMs often arose from non-ideal behaviors such as partial melting, supercooling, or extended temperature plateaus, which limit the predictability and stability of energy harvesting. In contrast, composite PCMs demonstrated faster thermal kinetics, more uniform phase transitions, and stable voltage profiles, despite reduced peak voltages (but very reasonable values and above the threshold of 10 mV) and total energy. The narrow-range composite excelled in rapid and consistent response, while the wider range composite provided sustained operation across a broader temperature span. Enhanced thermal conductivity and multiple nucleation sites in the composites facilitated more complete phase transitions and access to intermediate temperature ranges that pure PCMs often miss.

It is also important to note that the small-scale configuration of the prototype may have favored pure PCM unintentionally. The low PCM volume (3.5 mL) and the lack of additional insulation reduced the system's thermal inertia and allowed significant ambient buffering. Thus, suppressing the effective temperature difference across the TEG, even though the melting-point separation of the PCMs was large. Within this constrained geometry, the high conductivity of the copper wool accelerated heating and cooling in the composite, limiting thermal hysteresis and narrowing the harvesting window. It is highly likely that in systems with larger PCM volumes and more realistic heating and cooling cycles, the benefits of composite PCMs, especially their responsiveness and broader operational temperature range, would likely be even more pronounced. The experimental constraints used in this experiment were intentionally chosen to mimic natural thermal management scenarios, such as a miniature building-integrated system where precise temperature regulation and high-performance thermal insulation are impractical. The aim was to evaluate the system performance under realistic and relevant conditions by mimicking operational environments as closely as possible.

#### 4.2.3 Comparison to the existing literature

Only a few experimental studies have explored dual PCM-TEG configurations, most notably those by Atouei *et al.*^14^ and Yousefi *et al.*^18^ In these studies, externally imposed, high-intensity directional heat loads (ranging from 26 W to 100 W) were used to induce a high temperature on one side of the TEG, with the PCMs primarily serving as thermal buffers or extended heat sinks. Consequently, the temperature gradient across the TEG often exceeded 20 °C, resulting in high peak open-circuit voltages (0.75 V to 1.0 V) with maximum power outputs in the milliwatt range. The system performance of these studies is listed and compared to the present work in Table 2.

**Table 2:** Comparison of system performance and total energy yields between the presented dual PCM-TEG harvester and similar related studies

Study	System	Heat source	Peak voltage	Maximum power (*P*_max_)	Duration (min)	Energy yield (J)
Yousefi *et al.*^18^	PCM56/PCM36.5	Ceramic heater (26 W)	∼0.9–1 V	40.5–50 mW	108	158
Experimental	(Dual-sided copper foam)	Rectangular heat load	@ 26 °C			
Atouei *et al.*^14^	PCM65/PCM30	Electric heater (100 W)	0.9–0.95 V	∼168 mW	∼83	210
Experimental	(Pure PCM)	Transient heating	@ 30 °C			
Borhani *et al.*^17^	PCM69/PCM35	50 W	0.75 V	39 mW	∼26.6	18.83
Numerical	(Cold-side copper foam)	Periodic heat flux	@ 21.4 °C			
	PCM15/PCM32	Simulated ambient thermal cycling	57 mV	189.8 µW	109	446
	(Pure PCM)		@ 3.5 °C			
Present work	PCM15/PCM32	(TEC-driven copper plate)	∼36 mV	∼75.7 µW	131	304
Experimental	(Cu wool–GnP composite)		@ 3.5 °C Δ*T*			

A key difference lies in the energy harvesting duration. In the reported studies, operation was limited to approximately 80–100 minutes, as such high heat inputs are not continuously available. As a result, the total electrical energy generated ranged only between 150 and 200 J. In contrast, our system operates under an ultra-low-grade temperature difference (≤3.5 °C) under transient heating conditions, without any external directional heat source. Through the strategic use of phase transitions to generate an internal temperature gradient, the active energy harvesting duration is extended to up to 109 minutes for the pure PCM15/PCM32 system and up to 131 minutes for the thermally enhanced Cu wool–GnP composite.

Despite the lower absolute peak power, this extended-duration ambient harvesting enables the accumulation of higher total energy outputs (300–446 J) that are significantly higher than those of previously reported systems, even though they achieve higher peak voltages. This contrast is further emphasized by the numerical investigation of Borhani *et al.*,^17^ despite utilizing a 50 W periodic heat flux and cold-side copper foam enhancements to achieve 39 mW of peak power, their system's active harvesting duration was restricted to approximately 27 minutes, a total energy output of just 18.83 J.

Furthermore, numerical work by Meng *et al.*^41^ emphasized that dual PCM-TEG configurations with high-thermal-conductivity enhancements are environmentally robust and stable, even if the absolute peak output is lowered. Their findings indicate earlier phase initiation, shorter transition times, and enhanced voltage stability under thermal disturbances. These observations are consistent with the behavior of our Cu wool–GnP composite system, which sacrifices peak voltage in favor of stable, sustained voltage generation (32 mV on average) over an extended 131-minute harvesting period.

However, none of these aforementioned studies investigates a dual PCM-TEG configuration within the same application context as the present work. Unlike externally driven systems, our system operates without a dedicated heat source or heat sink, where the temperature gradient is generated internally through mismatched PCM phase change kinetics. This allows the system to harvest energy solely from ambient thermal variations, like day–night cycles, something not achievable with a TEG that lacks an imposed thermal gradient.

Thus, while earlier work highlights the benefits of dual PCM arrangements and enhanced heat transfer structures, the present work introduces a fundamentally different operating mechanism and application context. To date, there is a scarcity of data regarding dual PCM-TEG applications operating purely under low-temperature, low-grade ambient fluctuations without any external directional heating. This study addresses that gap by demonstrating that internally generated temperature gradients, driven by mismatched PCM phase-change behavior, can effectively enable energy harvesting from purely ambient variations.

## 5 Conclusions

This study demonstrates a novel operational concept of dual PCM-TEG configuration by showing that a temperature gradient can be generated internally, without any defined hot or cold side, solely through the mismatched phase-change kinetics of two PCMs. This mechanism enables energy harvesting under uniform, ambient-like thermal cycling, creating two distinct, repeatable thermal gradients within each thermal cycle, demonstrating a fundamentally different pathway for low-grade thermal energy conversion. The introduction of the hybrid composite with graphene nanoplatelets (GnP) and copper metal wool further strengthens the practicality of this concept. By achieving nearly 1 W m^−1^ K^−1^ thermal conductivity, the composite enables faster and more uniform heat transfer, offering clear advantages where response time, stability, or predictable voltage output are critical. Composite systems are suitable for time-sensitive or stability-critical applications, while pure PCMs are preferable when maximum energy yield is the priority. These contrasting behaviors outline a design framework in which PCM selection can be matched to the performance demands of specific applications.

A primary limitation of the present work noted was the small-scale prototype design and lack of robust insulation, which may have unintentionally suppressed the composite's full potential. Thus, future work will focus on design optimization needed to enhance thermal isolation (especially around TEG faces) and validation under real-world cycling conditions.

Overall, this work introduces a distinct operational concept of the dual PCM-TEG systems, and these insights are significant for designing self-powered or energy-autonomous devices for use in low-grade waste heat recovery systems or ambient thermal energy harvesting scenarios.

## Author contributions

Swathi Krishna Subhash—conceptualization, methodology, investigation, visualization, writing (original draft); Jesvin Jose—methodology, investigation; Peter Woias—writing (review & editing), supervision, conceptualization, project administration; Uwe Pelz—writing (review & editing), supervision, conceptualization, project administration.

## Conflicts of interest

There are no conflicts to declare.

## Supplementary Material

RA-016-D6RA02643E-s001

## Data Availability

The data supporting the findings of this study are included within the manuscript and its supplementary information (SI). The raw data associated with this manuscript is available from the corresponding author upon request. Supplementary information is available. See DOI: https://doi.org/10.1039/d6ra02643e.
