# Supplementary material for: Dual PCM integrated thermoelectric generator for harvesting energy from ambient temperature variations
Source: RSC Adv. 2026 Apr 21;16(23):20797–808. doi: 10.1039/d6ra02643e (PMC13097303; doi:10.1039/d6ra02643e)
Supplement: RA-016-D6RA02643E-s001 [file RA-016-D6RA02643E-s001.pdf]

Supplementary Information (SI) for RSC Advances.
This journal is © The Royal Society of Chemistry 2026

Supplementary Information for

"Dual PCM Integrated Thermoelectric Generator for Harvesting Energy from Ambient Temperature Variations"

Swathi Krishna Subhash[a,b], Jesvin Jose[a,b], Peter Woias[a,b] and Uwe Pelz[a,b]

[a] *Department of Microsystems Engineering – IMTEK, University of Freiburg (Germany)*

[b] *Cluster of Excellence livMatS @ FIT – Freiburg Center for Interactive Materials and Bioinspired Technologies, University of Freiburg (Germany)*

Microstructure and component analysis (SEM; FTIR)

The SEM images obtained of pure GnP, pure paraffin and paraffin-GnP (3 wt-%) composite with and without SDBS are shown in Figure S1(a)–(d). Graphene platelets exhibit a flaky, layered structure due to van-der-Waals interactions (Figure S1 (a)). Pure paraffin shows large, smooth layered structures with some darker edge regions likely from beam-induced melting (Figure S1(b)). Both composites retain layering but with rougher surfaces, indicating small GnP flakes embedded in the paraffin. The SDBS-containing sample is notably rougher and more disordered, suggesting better GnP integration. The inset in Figure S1 (d) shows GnP flakes engulfed by paraffin, with edge melting, indicating improved dispersion from the surfactant effect of SDBS.

FTIR spectra of PCM, GnP, and 3 wt% GnP composites (with/without SDBS) are shown in Figure S1(e), displaying C–H stretching and bending bands (600–4000 cm^{-1}) characteristic of aliphatic hydrocarbons and fatty acid esters. No peaks appear for GnP, and composites show no new peaks, indicating no chemical reactions. After 30 heating–cooling cycles, spectra remain unchanged, confirming material stability.

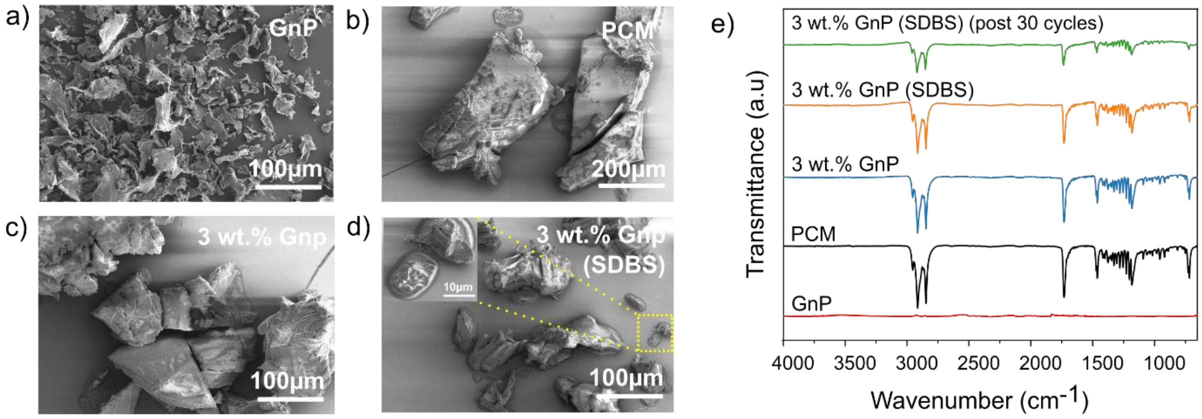

Figure S1. a) SEM image of pristine GnP showing the flaky morphology b) SEM image of pure PCM showing chunky and layered structure c) SEM image of PCM-GnP composite (3 wt-%) without SDBS d) SEM image of PCM-GnP composite (3 wt-%) with SDBS with inset showing likely GnP flakes engulfed by paraffin, with paraffin melting to the edges. e) FTIR spectra of GnP, PCM, PCM-GnP composite (3 wt-%) with and without SDBS and PCM-GnP composite (3 wt-%, with SDBS) post 30 thermal cycles, with heating and cooling between 0 °C and 60 °C.

Thermal properties (DSC; Thermal conductivity)

Table S1. DSC measurements: melting temperature range: onset temperature (T_i), melting temperature (T_m) and final temperature (T_f), latent heat: melting (ΔH_m) and solidifying (ΔH_s) and corresponding thermal conductivity

Sample	Melting range (° C)			Melting	Solidifying	Thermal conductivity ($W\ m^{-1}K^{-1}$)
	T_i	T_m	T_f	ΔH_m (Jg^{-1})	ΔH_s (Jg^{-1})	
PCM	36	41.9	44.3	185.5	-185.9	0.24 ± 0.01
PCM +3 wt-% GNP (without SDBS)	37	43	47.9	169.3	-169.4	0.44 ± 0.01
PCM +3 wt-% GNP (with SDBS)	35.5	38.8	40.4	170.5	-170.5	0.68 ± 0.01
Post 30 cycles DSC	35.6	39.1	40.6	164.6	-163.1	----

Dual-PCM TEG performance

Table S2: Summary of maximum ΔT and Voc (absolute value) from pure PCM and Composite Systems for PCM21/PCM29

Region	Process	pure PCM system		composite system	
		V_{oc} (mV)	ΔT (°C)	V_{oc} (mV)	ΔT (°C)
I	Melting of PCM21	19	1.1	31	1.8
II	Melting of PCM29	54	3.2	41	2.5
III	Solidification of PCM29	32	1.9	36	2.1
IV	Solidification of PCM21	30	1.8	34	2.1

Table S3: Summary of maximum ΔT and V_{oc} (absolute value) from pure PCM and Composite systems for PCM15/PCM32

Region	Process	pure PCM system		composite system	
		V_{oc} (mV)	ΔT (°C)	V_{oc} (mV)	ΔT (°C)
I	Melting of PCM15	21	1.3	32	1.7
II	Melting of PCM32	57	3.5	36	1.8
III	Solidification of PCM32	44	2.7	33	1.6
IV	Solidification of PCM15	--	1.85	5	0.3

References

1. S. A. Atouei, A. Rezania, A. Ranjbar and L. Rosendahl, *Energy*, 2018, 156, 311–318.
2. S. Borhani, M. Hosseini, R. Pakrouh, A. Ranjbar and A. Nourian, *Renewable Energy*, 2021, 168, 1122–1140.
3. J.-H. Meng, D.-Y. Gao, Y. Liu, K. Zhang and G. Lu, *Energy*, 2022, 245, 123332.
4. E. Yousefi, A. Abbas Nejad and N. Sayyar, *Applied Thermal Engineering*, 2023, 233, 121156.
